# ‘Is This the Workplace for Me?’ A Qualitative Study of Nursing Students' Construction of Workplace Attractiveness in Clinical Placement

**DOI:** 10.1002/nop2.70868

**Published:** 2026-09-30

**Authors:** Åsa Tjulin, Julia Cederlund, Åsa Chaikiat Ståhl, Emma Brulin, Bodil J. Landstad, Maritha Jacobsson, Maria Härgestam, Ann Rudman

**Affiliations:** ^1^ Department of Health Sciences Mid Sweden University Östersund Sweden; ^2^ Division of Psychology, Department of Clinical Neuroscience Karolinska Institutet Stockholm Sweden; ^3^ School of Health and Welfare, Department of Caring Sciences Dalarna University Falun Sweden; ^4^ Institute of Environmental Medicine Karolinska Institutet Stockholm Sweden; ^5^ Unit of Research Östersund Hospital, Region Jämtland Härjedalen Östersund Sweden; ^6^ Department of Social Work Uppsala University Uppsala Sweden; ^7^ Department of Nursing Umeå University, Region Västerbotten Umeå Sweden

**Keywords:** education, mentorship, nursing students, nursing workforce, organisational culture, professional development, supportive work environment, transition to worklife, workplace attractiveness

## Abstract

**Aim:**

To explore how nursing students perceive and construct the notion of workplace attractiveness in healthcare during clinical placements.

**Design:**

Qualitative explorative research design.

**Method:**

Thirteen final‐year undergraduate nursing students from four Swedish universities participated in seven semi‐structured interview sessions between February and May 2025: three individual interviews and four small‐group interviews involving two to three participants. An interview guide, organised around main themes, directed the conversations. Interviews were digitally recorded and transcribed verbatim. Data analysis was performed using reflexive inductive thematic analysis, characterised by an iterative, flexible, and interpretive approach rooted in a constructionist epistemology.

**Results:**

Clinical placements shaped nursing students' perceptions of workplace attractiveness, forming the basis for future career choices. The overarching theme, *Looking Ahead*, captured how students evaluated healthcare as a potential workplace while envisioning their professional future. Four interrelated themes emerged: *Becoming*—gaining confidence and identity as a nurse through hands‐on experience; *Belonging*—the importance of relationships and social climate for inclusion; *Balancing*—concerns about managing work‐life integration and sustainable conditions; and *Beyond*—seeking opportunities for continued growth and development. Together, these themes illustrate workplace attractiveness as relational and temporal, emerging from immediate experiences and long‐term aspirations.

**Conclusions:**

Workplace attractiveness is a dynamic, relational process shaped by interactions between individuals and organisations, not a fixed attribute. It evolves through everyday practices, social identity, and belonging. Effective strategies must address relational and symbolic dimensions, fostering inclusion, identity formation, and meaningful participation alongside structural improvements.

**Implications for the Profession and/or Patient Care:**

Understanding workplace attractiveness as a dynamic process highlights the need for healthcare organisations to prioritise supportive onboarding, inclusive social climates, and opportunities for professional growth. Strengthening belonging, identity formation, and sustainable working conditions can enhance nurse retention, reduce turnover, and ultimately improve continuity and quality of patient care.

**No Patient or Public Contribution:**

This study did not include patient or public involvement in its design, conduct, reporting, or dissemination since it focused on nursing students' perceptions during clinical training rather than on patient experiences.

**Impact:**

- Problem addressed: Limited understanding of how nursing students construct perceptions of workplace attractiveness during clinical placements and how these perceptions influence future career choices.
- Main findings: Workplace attractiveness emerged as a dynamic, relational process shaped by five themes: *Looking Ahead*, *Becoming*, *Belonging*, *Balancing*, and *Beyond*. Clinical placements were pivotal in forming these perceptions.
- Impact: The findings inform healthcare organisations, educators, and policymakers to strengthen onboarding processes, foster a sense of belonging, and promote sustainable working conditions. This can improve nurse recruitment, retention, and ultimately the quality and continuity of patient care.
- Highlights the of clinical placements in shaping nursing students' perceptions of workplace attractiveness, which can inform global strategies for improving nurse recruitment and retention.
- Introduces a relational and dynamic understanding of workplace attractiveness, emphasising identity formation, belonging, and professional growth—key factors for creating supportive and sustainable healthcare environments worldwide.
- Provides actionable insights for healthcare organisations and educators, encouraging inclusive onboarding, social integration, and career development opportunities to enhance workforce stability and patient care quality globally.

## 1 Introduction

The recruitment and retention of nurses remain persistent challenges for healthcare systems worldwide, affecting workforce capacity and continuity of care (World Health Organization [WHO] 2020). International policy and workforce reports continue to emphasise nursing shortages, turnover and the need for sustained investments in nursing education, working conditions, and leadership to support workforce sustainability (WHO 2020; Sharplin et al. 2025). In parallel, workplace attractiveness has gained attention in research and practice as a concept used to understand factors that support recruitment, retention, and long‐term workforce stability in healthcare (Karjalainen et al. 2025; Koch‐Rogge and Westermann 2021; Salminen‐Tuomaala and Herttuala 2024). Workplace attractiveness refers to the perceived desirability of a workplace as a current or prospective place of employment. Previous healthcare research suggests that such evaluations may concern both organisational and material conditions and experiential aspects of work (Karjalainen et al. 2025; Koch‐Rogge and Westermann 2021). Reviews of organisational attractiveness in healthcare primarily synthesise evidence from practising nurses and employed staff, leaving student perspectives less visible in the attractiveness literature (Karjalainen et al. 2025). There is comparatively limited empirical work examining how nursing students, prior to entering the labour market, construct what makes a workplace attractive during clinical placement (Squire et al. 2024). At the same time, nursing education research has tended to focus on learning outcomes and placement experiences without necessarily framing these experiences explicitly in terms of workplace attractiveness as a concept relevant to recruitment and retention (Levett‐Jones and Lathlean 2008; Squire et al. 2024). This combination suggests an important gap at the intersection of workforce and nursing research: how students interpret clinical placements as potential future workplaces and how they articulate what makes a workplace attractive (Karjalainen et al. 2025; Squire et al. 2024).

## 2 Background

Research on organisational or workplace attractiveness in healthcare indicates that determinants extend beyond structural conditions and include workplace climate, learning opportunities, and social integration (Karjalainen et al. 2025; Hörberg et al. 2023). Although organisational attractiveness is often discussed in relation to working conditions and organisational support, evidence from nursing research shows that relational and cultural dimensions, such as inclusive team climates and supportive environments, are central to how workplaces are experienced and evaluated (Hörberg et al. 2023; Karjalainen et al. 2025). This aligns with research that conceptualises attractiveness in healthcare as involving both material and experiential aspects of work, including how workplaces enable professional development and sustainable working lives (Karjalainen et al. 2025; Koch‐Rogge and Westermann 2021).

Although organisational attractiveness is often examined in relation to employed staff, research suggests that orientations toward work, organisational fit, and professional sustainability begin to take shape earlier in professional trajectories. In this sense, workplace attractiveness can be understood as intertwined with processes of transition and organisational socialisation that precede stable employment. Empirical research on the transition from nursing education to professional practice demonstrates that this phase is strongly influenced by organisational conditions (Rudman and Gustavsson 2012). Studies show that burnout symptoms increase across education, and that higher levels of exhaustion and disengagement are associated with poorer occupational preparedness, reduced mastery of clinical tasks, and more limited use of evidence‐based practice 1 year post‐graduation (Rudman and Gustavsson 2012). Research further indicates that early socialisation experiences are associated with stress, strain, and early career burnout, underscoring the significance of organisational support and relational conditions during early professional phases (Frögéli et al. 2022).

Organisational socialisation theory conceptualises successful adaptation as dependent on resources that reduce uncertainty and foster competence and belonging (Saks et al. 2007; Bauer et al. 2007). Three core processes, task mastery, role clarity, and social acceptance, are central to this adjustment. Task mastery supports confidence and perceived control, role clarity reduces ambiguity, and social acceptance promotes inclusion and belonging. Together, these processes are associated with well‐being, performance, and retention, and have been linked to job satisfaction, organisational commitment, and professional success (Saks et al. 2007; Bauer et al. 2007). Qualitative studies on nurses' intentions to leave the profession further illustrate how motivational patterns and turnover‐related reasoning are connected to workplace experiences, including perceived support and organisational climate (Hörberg et al. 2023). Taken together, these findings support the view that perceptions relevant to workplace attractiveness and retention can begin to form early in nursing education and professional trajectories, even before individuals enter stable employment (Rudman et al. 2014; Frögéli et al. 2022; Karjalainen et al. 2025; Chen et al. 2025).

For nursing students, these processes of socialisation and early evaluation of work do not begin at the point of employment, but during clinical placements, which often constitute their first sustained encounters with healthcare organisations as workplaces. Clinical placements are widely recognised as formative spaces where students develop clinical competence, professional confidence, and a sense of belonging (Levett‐Jones and Lathlean 2008; Squire et al. 2024). Empirical research on clinical learning environments shows that students' learning and professional development are shaped by supervision, relational support, and opportunities to participate meaningfully in practice (Levett‐Jones and Lathlean 2008). Further, nursing education research emphasises that belongingness during placements is closely tied to learning and identity development, highlighting the importance of inclusive learning climates and supportive relationships in clinical education (Squire et al. 2024). These findings position clinical placements not only as educational contexts but also as environments where students encounter workplace cultures, team dynamics, and organisational practices that may influence how healthcare work and workplaces are evaluated (Squire et al. 2024).

Against this backdrop, there is a need to better understand how workplace attractiveness is formed during the socialisation processes of clinical placements before students become part of the workforce, because placements constitute a central arena where students develop expectations, preferences, and professional orientations toward future work (Squire et al. 2024). A deeper understanding of how nursing students perceive and construct workplace attractiveness during clinical placements is therefore needed to inform both healthcare organisations and educational institutions. Such knowledge can contribute to efforts to improve clinical learning environments, strengthen early professional socialisation, and support sustainable workforce development in healthcare (WHO 2020; Karjalainen et al. 2025; Squire et al. 2024).

## 3 The Study

### 3.1 Aim and Research Questions

The study aims to explore how nursing students perceive and construct the notion of workplace attractiveness in healthcare during clinical placements.

The study was guided by the following research questions:

- How do nursing students describe experiences that shape their perceptions of workplace attractiveness during clinical placements?
- What aspects of clinical placements influence students' views of healthcare workplaces as potential future places of employment?

## 4 Methods

### 4.1 Design

We conducted an explorative qualitative study using semi‐structured interviews to discover how undergraduate nursing students construct workplace attractiveness during clinical placements. The study employed reflexive thematic analysis (Braun and Clarke 2021) with an inductive, data‐driven orientation situated within a constructionist epistemology. This approach recognises meaning‐making as co‐produced through participants' accounts and the interpretative work of researchers, which makes it particularly suitable for exploring students' experiences within relational and placement‐based contexts.

### 4.2 Setting, Participants, and Data Collection

This study focused on undergraduate nursing programmes at four Swedish universities (labelled A‐D) across the country. The Swedish nursing programme is a 3‐year, full‐time bachelor's degree comprising 180 European Credit Transfer and Accumulation System (ECTS) credits and combining theoretical coursework with clinical practice. In line with the European Union professional qualifications directive's requirement that at least 50% of the programme be clinically oriented, most Swedish universities include approximately 43 weeks of clinical placements. After graduation, students can apply for a nursing licence through the Swedish National Board of Health and Welfare and begin working as registered nurses.

Data collection occurred from February to May 2025 and involved 13 final‐year nursing students: four from University A, two from University B, four from University C, and three from University D. The participant group comprised nine women and four men. The universities served as recruitment settings rather than as separate analytical cases, and the analysis was conducted across the complete dataset. All eligible students were initially invited to participate in small‐group interviews via Zoom. Students who were unable to attend the scheduled group sessions were offered individual interviews at alternative times. Three individual interviews and four small‐group interviews involving two to three students. The inclusion of both formats therefore arose primarily from participant availability rather than from a planned comparison of interview modalities.

The formats generated somewhat different interactional conditions. Individual interviews allowed participants to develop personal accounts without co‐participants present, whereas the small groups enabled participants to prompt, elaborate on, and qualify shared experiences. Group interaction may also have encouraged consensus or constrained disclosure. Participants' accounts referred to clinical placements across hospital wards, primary care, emergency departments, ambulance services, and other healthcare settings. Detailed information about the duration of individual placements and the specific supervision or preceptorship models used at each placement site was not collected systematically as participant background data. These characteristics could therefore not be described consistently at the individual or site level. The same interview guide was used across formats, and all interviews were analysed as one dataset while attending to the interactional context in which accounts were produced.

Most interviews were conducted by two researchers: one senior qualitative researcher and one doctoral student in nursing. Their joint presence supported attention to group dynamics and thorough exploration of the interview guide. One individual interview was conducted by the senior researcher alone. Because neither interviewer had taught or supervised the students, relational dependency and influence were minimised.

Interviews lasted on average around 80 min. An interview guide with open‐ended questions encouraged participants to reflect on experiences of clinical placements, defining features of attractive workplaces, and perceptions of future employment settings. All interviews were audio‐recorded and transcribed verbatim. Field notes were written immediately after each interview to document contextual details and early analytic impressions. Data collection and preliminary analysis proceeded in parallel. Recruitment concluded when additional interviews no longer contributed perspectives relevant to the study aim.

### 4.3 Analysis

Data were analysed using Braun and Clarke's (2021) reflexive thematic analysis, with an inductive orientation situated within a constructionist epistemology. The analysis recognised the active and interpretative role of the researchers in generating meaning and developing themes. Three authors (Å.T., A.R., and J.C.) engaged collaboratively and recursively in data familiarisation, coding, and theme development. The transcripts were read repeatedly, and initial codes were revisited and refined as the researchers' engagement with the dataset deepened. Coding was treated as an open and evolving interpretative process rather than as the application of a fixed coding frame.

Candidate themes and subthemes were developed through an interpretative examination of patterns of shared meaning across the coded material, with attention to how these patterns addressed the research questions. Analytic discussions within the research team were used to question, extend, and refine interpretations rather than to establish coding agreement or verify a single correct reading of the data. Through this recursive process, the meaning, scope, boundaries, and relationships of the candidate themes and subthemes were further developed and refined.

The analysis resulted in four themes; Becoming, Belonging, Balancing, and Beyond, and their associated subthemes: A safe introduction to nursing practice; It is the co‐workers who matter; Lots of aspects must align; and A place to grow. Together, the themes and subthemes contributed to the overarching thematic pattern Looking ahead—workplace attractiveness as a socialisation process. Figure 1 presents the final thematic structure and the relationships between the overarching theme, the four themes, and their associated subthemes. It is not intended to represent a fixed or linear progression from individual codes to themes. The final analysis reflects a synthesis of inductive engagement with participants' accounts and the researchers' situated interpretative work.

**FIGURE 1:**
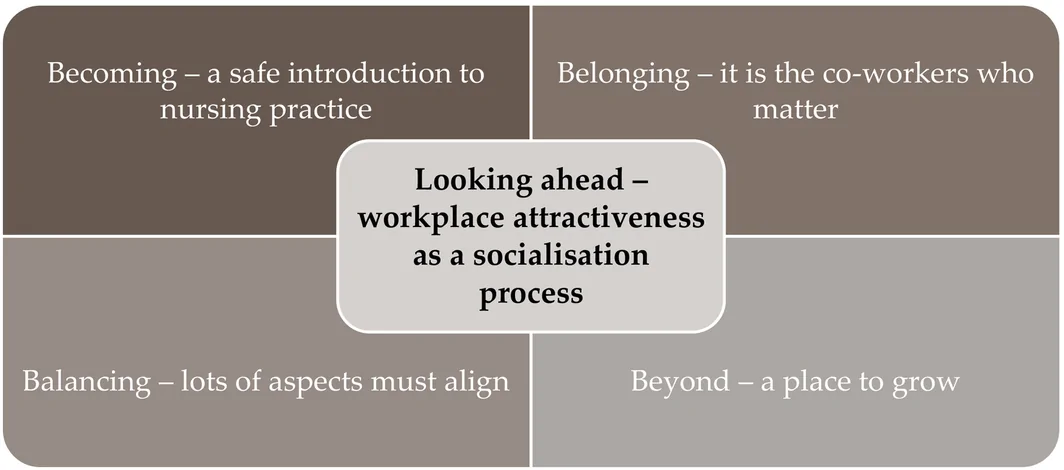
Thematic analytical model with overarching theme and themes.

### 4.4 Reflexivity and Trustworthiness

Reflexivity was integral throughout the study. Several members of the research team have backgrounds in health sciences and nursing, and long‐standing experience in healthcare education. Recognising that such professional proximity can both enable and constrain interpretative work, the team engaged in continuous reflexive dialogue about assumptions, preunderstandings, and how disciplinary lenses might shape meaning‐making. Reflexive memos and analytic conversations supported transparency in these deliberations. These practices were not intended to eliminate researcher subjectivity, but to make its role in the co‐construction of the analysis more visible.

Reflexive openness was supported through repeated engagement with the dataset, evolving coding, reflexive memo writing, and critical analytical dialogue within the research team. Collaborative analysis was used to deepen and challenge interpretations rather than to establish coding consensus or verify a single correct reading of the data (Braun and Clarke 2024). Verbatim extracts are presented in the findings to demonstrate the grounding of interpretations in students' accounts and to ensure analytic credibility.

### 4.5 Ethical Considerations

Ethical approval for this study was obtained from the Swedish Ethical Review Authority (Dnr 2024‐05983‐01). Potential participants received written information about the study as part of the initial invitation. Study invitations prepared by the research team were distributed through programme and course coordinators at each university via direct email and course learning platforms. The invitations provided written information about the study and contact details for a member of the research team. They were informed that they could contact the lead researcher by email or telephone with questions about the study or their participation and were again given an opportunity to ask questions before the interview. Students who were interested in participating contacted the research team directly.

Written informed consent was obtained before the interview, and verbal consent was reconfirmed at the beginning of each interview session. Participants were informed, both in writing and verbally, that participation was voluntary and that they could discontinue their participation at any time.

For the small‐group interviews, participants were informed that the research team would protect confidentiality in the handling and reporting of the research material, but that confidentiality between group members could not be guaranteed. Participants were therefore asked to respect one another's privacy and not to disclose outside the interview what other participants had shared. In reporting the findings, quotations were attributed to the interview group identifiers rather than participants' as individuals. As a token of appreciation for their time, participants received a gift card restricted to the purchase of one breakfast meal at a national food‐service chain.

## 5 Findings

The nursing students' experiences during clinical placement formed the foundation for how they perceived and constructed the notion of workplace attractiveness in healthcare. The clinical placements were where the students first became acquainted with healthcare as a workplace and where they learned the profession. They were not only learning environments but also arenas for evaluating potential employers. The first theme emphasises students' experiences of *becoming nurses* during clinical placements. The second theme underscores the importance of *belonging*, highlighting how relationships and the social climate shape inclusion and integration. The third theme reflects students' concerns about *balancing* work, personal life, and maintaining sustainable working conditions. The fourth theme looks *beyond* focusing on professional growth, where opportunities for continued development become central. Taken together, these themes illustrate the overarching theme of *looking ahead—attractiveness in socialisation processes*, where workplace attractiveness was constructed as relational and temporal, emerging in the interplay between students' immediate experiences and their visions of the future (Figure 1).

### 5.1 Overarching Theme: Looking Ahead—Workplace Attractiveness as a Socialisation Process

Workplace attractiveness as a socialisation process encompasses phases of becoming, belonging, balancing, and moving beyond, which occur during nursing students' clinical placements. Together, the identified themes constructed an experience of workplace attractiveness as something lived, relational, and context‐dependent. Rather than being a fixed attribute, attractive workplaces emerged through the interaction between students' needs, aspirations, workplace socialisation processes, and organisational practices. The sense of attractiveness formed, or lost, during clinical placements can profoundly shape students' outlook on their professional future. Workplace attractiveness was about balancing structure and flexibility, receiving a proper introduction, and feeling secure within a social context. The students perceived workplace attractiveness not primarily as a place, a promise, or a strategy, but as a sense of direction toward something meaningful. Within this experience reside both security and uncertainty, determination and hesitation about the future. Thus, workplace attractiveness should be understood not as a finalised solution, but as an ongoing, lived practice.

### 5.2 Becoming: A Safe Introduction to Nursing Practice

This theme concerns being welcomed into and included in the clinical setting, as well as the quality of interpersonal relationships with the supervisor, which often proves decisive in determining what makes a workplace attractive. The students described clinical placement as not only a means to practice their professional skills but also to develop as professionals. When this falls into place, they perceive the workplace as an attractive option. The students described it as not just an educational climate, but also a work culture where newcomers were greeted with anticipation rather than hesitation. It was not just about ‘doing things right’ but about being recognised as a future colleague and stepping into a context where their developmental potential was trusted. Experiences of being treated as future colleagues rather than as temporary burdens were highlighted as decisive in how a workplace was perceived. ‘I received no introduction, no schedule—I didn't even know whom I was supposed to shadow. I just thought: I'm never coming back here’. (Student, FG3).

The supervisor emerged in the descriptions as a key person, as a role model and guide, both socially within the workplace and professionally within the role, but sometimes also as a barrier. When supervisors took time, responded warmly, and created security, the students' self‐confidence quickly grew. Supervisors who were described positively were those who took the time to explain, ask questions, and continually create learning situations that challenged students at an individually tailored level, expressing confidence in students' abilities. One participant said: ‘My supervisor didn't see me as a student but as a colleague in training—that made all the difference’. (Student, FG 2). Where supervision was lacking, students describe feelings of insecurity, passivity, and sometimes abandonment. In such situations, the supervisor was described as overwhelmed, uninterested, or unprepared for the supervisory role. The students also reflected on having had newly graduated nurses as supervisors, which often led to reduced learning opportunities, as these supervisors themselves were not fully comfortable with workplace routines and tasks. In these cases, uncertainty arose regarding one's role and position in the team. Supervision, thus, cannot be seen merely as a learning tool; students also emphasised its social relational importance.

A recurring description among students was that attractive workplaces were those where there exists time, structure, and a willingness to welcome newcomers. Where introductions, feedback, and presence have worked well, students described feeling ‘in good hands’. Conversely, when no one knew who they were and silence replaced dialogue, part of their sense of security disappeared. One student described a neurology ward where she received a list of words and concepts, a group introduction, and a clear schedule, making her feel secure from the start. However, where the introduction was lacking, or the student's role was down‐prioritised, learning turned into hesitation. This was not solely about uncertainty in clinical skills, but about a broader feeling of being unwanted, not knowing one's place, and not understanding expectations. It concerned a lack of access to informal knowledge and a feeling of disempowerment to act. One student explained: ‘Mostly, I just wanted the weeks to pass. But that was because it was far too slow‐paced for me, and the staff there were … well, they were ready to retire. So they were a bit bitter …’ (Student, FG1). This reinforced feelings of exclusion and being a passive observer rather than an active learner. A reaction among students who did not feel well‐received was that they did not wish to return, regardless of how engaging the tasks were. These experiences indicated that attractiveness in this phase was constructed through relational security, structure, and validation.

### 5.3 Belonging: It Is the Co‐Workers Who Matter

The analysis revealed that the most enduring impressions of a workplace's attractiveness rarely revolve around workload or patient cases. Instead, they revolved around co‐workers: the person who asked how you were feeling, ensured time for a coffee break, or contributed to moments when the team truly felt like a team. Attractiveness was cultivated through interpersonal interaction, underscoring the need to understand how relational qualities are fostered in the workplace.

Students emphasised the importance of the social climate, noting elements such as tone, jargon, and interactions around the lunch table. Workplaces where students felt included, noticed, and where communication was open were described with warmth. Teams that were frequently highlighted as especially appealing were those where mutual support was evident, collaboration was prioritised, and questions were welcomed, as described by one student: ‘I also think that it's not just the work tasks that matter. I think it depends a great deal on the team you join and the manager you have. If you have a good manager and a good team, then for me personally, the other things are somewhat secondary’. (Student, IP 2). However, the analysis also revealed descriptions of less attractive workplaces and their drawbacks, particularly those marked by interpersonal friction, subtle exclusion, and a lack of personal engagement. These environments were often described as dominated by complaints and gossip, and where students felt excluded during lunch breaks. Such experiences created emotional distance from the specific workplace. As one student described it: ‘It wasn't the patients who discouraged me—it was the colleagues’. (Student, FG1).

The students constructed workplace attractiveness within the local culture and day‐to‐day experience, including how colleagues collaborate, communicate, and show care in practice. What was communicated in social interactions, such as a greeting or an inclusive comment, was described as of substantial significance. Students discussed team spirit, support, and community, and foundational elements, such as job satisfaction and a sense of being needed and belonging, play a central role.

### 5.4 Balancing: Lots of Aspects Must Align

Clinical placements that offered an experience of balance, where factors such as geographical location, working hours, and organisational support contributed to a sustainable life situation, enhanced workplace attractiveness. Students described specific prerequisites for a well‐functioning workplace, including clear organisational structures, a manageable workload, and supportive social relationships that foster both job satisfaction and learning.

The students experienced workplaces as attractive when they could reconcile work with their personal lives. Clinical placements were described as eye‐opening, particularly regarding the diverse scheduling practices across various healthcare settings (e.g., primary care centres, hospital wards, emergency departments, ambulance services). Although some schedules worked smoothly, others prompted reflection on how to balance work hours with leisure time and family life.

Students discussed placements where there was a rhythm to the workday and a balanced workload, allowing for time for breaks, recovery, and reflection with their supervisor. They talked positively about placements where the workday had a natural rhythm and the workload was manageable. As one student noted in one placement: ‘They also have really good routines. The way the whole ward operates is very structured, and I like that. It's like … they know when they're supposed to come in, they know exactly what time certain things are done. I like that kind of structure too’. (Student, FG 4). Workplace attractiveness was linked to simple yet significant moments: sharing a coffee break, enjoying a calm lunch, or simply having space to breathe during the workday. When the pace was reasonable and responsibilities fairly distributed, the students experienced a sense of camaraderie. It was in these moments that laughter occurred, and someone genuinely asked how one was doing.

The geographical dimension was also highlighted as an essential aspect of achieving balance for the students. The ability to work close to home and family was considered vital for both emotional well‐being and logistical sustainability. Some students, for example, expressed a desire to return to or remain in their rural hometowns, without compromising professional opportunities. Students with children or strong leisure interests were particularly attuned to the conditions necessary for long‐term work sustainability. As one student expressed, ‘It became easier to breathe when I realised there are jobs Monday to Friday. I thought all healthcare work was evenings and weekends’. (Student, FG2).

### 5.5 Beyond: A Place to Grow

Workplace attractiveness was experienced as closely linked to optimism about the future and the recognition that the nursing profession offers breadth and opportunities for lifelong learning. This was reflected in a desire to grow; ‘…so that you don't just get stuck in the daily grind and become another number in the region's workforce statistics, but instead have the opportunity to shape your own path and are given the tools and support to do so’ (Student, IP 1), whether by changing direction, deepening expertise, or broadening skills and competencies. At the same time, students acknowledged that opportunities for personal and professional development were influenced by organisational conditions, emphasising that employers were expected to actively support and facilitate this journey.

The students recounted experiences of being directly or subtly recruited during clinical placements, sometimes after only a few days on‐site. Phrases such as ‘You're welcome back later’, ‘We're looking for people’, or inquiries about post‐graduation plans were described as common. Although some students interpreted these gestures as affirming and welcoming signals that they were competent and desirable, others experienced them as reductive, feeling viewed more as a staffing resource than as learners. ‘It felt like they mainly needed staff—not that they cared about how I was doing as a student’. (Student, FG3). This uncovered a tension between learning and recruitment. When students were regarded as future colleagues, a sense of belonging developed; however, when staffing needs outweighed supervision and education, uncertainty and doubt arose. Students perceived workplace attractiveness as based on real opportunities for growth within a safe and inclusive space, where they could advance professionally and truly feel valued as part of a community.

The students reflected on the differences between permanent staff and temporary agency nurses, expressing a mix of fascination and hesitation. Temporary positions were often associated with greater freedom, higher wages, and increased control over scheduling. At the same time, they were perceived as signalling a lack of loyalty, fragmented teams, diminished continuity of care, and the potential for growth and development as a nurse. Although students understood the appeal of temporary work, they also described feeling uneasy entering departments where team members were unfamiliar with one another. This ambivalence reveals a compelling contrast: development and mobility were attractive, but not at the expense of social cohesion and caregiving responsibility. The desire for flexibility coexists with a need for stability, trust, and shared professional identity and growth, elements that were essential for both safe care and a sense of belonging.

## 6 Discussion

This study aimed to explore how nursing students perceive and construct workplace attractiveness in healthcare during clinical placements. The findings show that workplace attractiveness, as constructed by nursing students during clinical placements, cannot be understood as a fixed set of organisational features. Rather, attractiveness emerged as a dynamic and relational process, shaped through students' positioning, inclusion, and participation in everyday clinical practice. By drawing on interviews with nursing students, the study provides insight into how evaluations of future workplaces emerge already during clinical placements. Students' assessments of attractiveness were thus grounded in immediate experiences while simultaneously oriented toward imagined professional futures. This interpretation resonates with previous research showing that educational and clinical environments shape nursing students' professional commitment and future orientation (Chen et al. 2025). By foregrounding nursing students' perspectives, the present study extends existing research on organisational attractiveness in healthcare, which has predominantly focused on employed staff (Karjalainen et al. 2025).

To deepen the interpretation of the findings, the discussion draws on theoretical perspectives that foreground social relations, identity processes, and participation in practice. These perspectives are not used as deductive or causal explanatory frameworks imposed on the empirical material. Rather, they function as sensitising concepts that support a theoretically informed interpretation of patterns already identified in the analysis (Braun and Clarke 2021).

The discussion is organised around three interrelated analytical foci: social relational practices in clinical settings, processes of emerging professional identity and legitimacy, and how access to learning and participation shapes students' orientations toward work. This progression reflects how students' experiences unfold during clinical placements, moving from immediate interactions in everyday practice, through developing senses of belonging and recognition, to more tentative, experience‐based assessments of whether a workplace feels meaningful and possible to imagine returning to in the future.

### 6.1 Social Relational Understanding of Workplace Attractiveness

The findings demonstrate that nursing students constructed workplace attractiveness primarily through everyday relational experiences within the immediate clinical environment. Rather than referring to formal organisational structures or articulated policies, students' accounts centred on how they were met, recognised, and included in daily interactions. This aligns with research highlighting that experiences of inclusion and relational support are central to how healthcare workplaces are perceived and evaluated, particularly in contexts of professional socialisation (Levett‐Jones and Lathlean 2008). Workplace attractiveness thus emerged in the micro‐settings of clinical work, where seemingly ordinary encounters, such as being acknowledged, invited into conversations, or treated as a legitimate participant, carried decisive meaning.

Previous research has shown that early experiences of organisational socialisation influence long‐term engagement, stress, and intentions to remain in the profession (Bauer et al. 2007; Frögéli et al. 2022; Chen et al. 2025). Extending this work, the present findings indicate that students used relational experiences during clinical placements to assess whether a workplace appeared compatible with a sustainable professional life over time. Workplace attractiveness was thus constructed as a future‐oriented judgement, grounded in present interactions yet oriented toward anticipated professional trajectories.

Belonging and being part of a team emerged as central mechanisms for attractiveness. When students experienced relational inclusion, they reported increased confidence and a stronger inclination to envision themselves working within the organisation. This resonates with social belonging theory, which emphasises that feelings of acceptance and emotional safety are foundational for motivation, engagement, and meaning‐making (Baumeister and Leary 1995; Walton and Cohen 2007). Conversely, experiences of exclusion or marginal positioning generated what has been described as belonging uncertainty, which has been shown to undermine learning, confidence, and persistence in clinical education (Levett‐Jones and Lathlean 2008).

Taken together, these findings suggest that workplace attractiveness in clinical education cannot be understood independently of relational inclusion in everyday practice during clinical placement. Rather than being an inherent attribute of the workplace, attractiveness was actively constructed through students' experiences of whether they were socially and emotionally anchored in the clinical environment in ways that supported learning, identity development, and future participation. This positions social relational practices as central mechanisms through which workplace attractiveness takes shape and through which students begin to orient themselves toward or away from particular organisational contexts.

### 6.2 Social Identity and the Possibility of Becoming a Future Colleague

The findings further indicate that workplace attractiveness was closely linked to whether nursing students were positioned as future colleagues rather than solely as temporary learners. Experiences of being entrusted with responsibility, invited to contribute to patient care, and addressed as legitimate members of the team carried particular significance, as they signalled recognition not only of students' current learning efforts but of their emerging professional identity. This aligns with research suggesting that early recognition and inclusion are central to professional socialisation and identity development in healthcare contexts (Levett‐Jones and Lathlean 2008; Saks et al. 2007). Although belonging captures students' experiences of emotional and relational inclusion as a fundamental aspect of feeling connected at work (Levett‐Jones and Lathlean 2008; Squire et al. 2024), a social identity perspective allows these experiences to be understood in relation to a specific professional context. From this perspective, interactions of inclusion and recognition also signal legitimacy, group membership, and the possibility of becoming a recognised professional insider within the nursing profession (Ashforth and Mael 1989).

In these situations, workplace attractiveness was constructed less in relation to specific tasks and more in relation to whether the workplace acknowledged students' potential to become nurses within that organisational context. From a social identity perspective, these experiences can be understood as moments of shifting self‐categorisation, in which students moved from peripheral positions toward identification with the professional in‐group (Ashforth and Mael 1989). Being treated as a colleague provided symbolic and relational confirmation of students' legitimacy as future members of the profession, facilitating this transition. Consistent with social identity theory, attractiveness thus functioned as an assessment of whether the workplace enabled access to a professional identity that students could realistically inhabit and sustain over time (Ashforth and Mael 1989; Heide and Schröder 2024). Conversely, workplaces characterised by high staff turnover, reliance on temporary staffing, or fragmented teams were often met with scepticism. Such environments were perceived as offering limited opportunities for stable identification and long‐term belonging, thereby constraining the possibility of identity consolidation. Previous research has shown that group stability, continuity, and shared norms are critical conditions for identification and commitment within professional communities (Ashforth and Mael 1989). For nursing students, such conditions shaped whether it was possible to imagine oneself as a future member of the workplace, particularly in contrast to settings characterised by high staff turnover or fragmented teams.

Taken together, these findings suggest that workplace attractiveness during clinical education is deeply intertwined with identity development. Rather than being a response to isolated organisational features, attractiveness was constructed as a judgement about whether a workplace could support students' transition from learners to legitimate participants in the nursing profession. This positions access to social identity and recognition as central mechanisms through which workplace attractiveness is constructed and negotiated during clinical education.

### 6.3 Workplace Learning, Participation, and the Construction of Attractiveness

The findings further indicate that workplace attractiveness was closely linked to how learning and participation were organised and supported in clinical placements. Attractive workplaces were not primarily defined by formal educational structures, but by how students were gradually introduced to routines, supported by engaged supervisors, and entrusted with meaningful responsibilities. Such arrangements created a sense of direction and continuity in learning, signalling that students were moving along a recognisable developmental trajectory. This aligns with research emphasising that effective workplace learning depends not only on access to learning opportunities, but on how participation is structured and supported in everyday practice (Billett 2016). Learning involves gaining access to the social and cultural practices of the workplace, where participation is progressively negotiated and legitimised rather than automatically granted (Billett 2016). In line with previous research on organisational socialisation, students' opportunities to learn were shaped not by individual motivation alone, but by how their presence was recognised, guided, and embedded within everyday work practices (Saks et al. 2007; Bauer et al. 2007; Frögéli et al. 2022). When onboarding was limited, supervision was inconsistent, or learning was left largely to individual initiative, students experienced the workplace as fragmented and difficult to navigate, which diminished its perceived attractiveness.

Importantly, access to the guided participation functioned as a central mechanism through which students evaluated workplaces. When students were invited to move from peripheral observation toward more active participation, the workplace was experienced as a space for growth and professional development. Conversely, when participation was restricted or unevenly distributed, learning trajectories stalled, and workplace attractiveness diminished. In line with organisational socialisation research, these learning arrangements were closely tied to social acceptance, as students' access to participation depended on being recognised as legitimate contributors in everyday practice (Saks et al. 2007; Bauer et al. 2007). These findings resonate with research showing that structured support and relational mentoring are critical for sustaining engagement during education and early career transitions (Frögéli et al. 2022; Chen et al. 2025).

Although previous studies have highlighted supportive environments and development opportunities as key factors for employed nurses (Karjalainen et al. 2025; Hörberg et al. 2023), the present findings show that students already engage in similar evaluative processes during clinical placements. However, these evaluations were characterised by negotiation and ambivalence, as students balanced professional aspirations with concerns about continuity, workload, and long‐term well‐being (Karjalainen et al. 2025; Hörberg et al. 2023; Sharplin et al. 2025). Workplace attractiveness thus emerged not as a static attribute, but as a contingent judgement about whether a workplace enabled meaningful learning, progression, and participation in practice during clinical placement.

### 6.4 Limitations

The findings are contextually situated, and their transferability depends on the extent to which other settings may share relevant features of Swedish nursing education, clinical placement organisation, supervision practices, and healthcare labour‐market conditions. The sample consisted of final‐year nursing students from four Swedish universities. Educational structures, professional regulation, workplace cultures, and the organisation of clinical learning may differ across national and cultural contexts. Readers should therefore consider both similarities and differences when assessing the relevance of the findings to other educational systems, healthcare settings, and stages of nursing education. Because all participants were approaching graduation, the findings may particularly reflect concerns and expectations associated with the transition into professional practice and may differ from those of students earlier in their education.

All interviews were conducted via Zoom. This enabled participation across geographically dispersed institutions but may have affected rapport, access to non‐verbal cues, turn‐taking, and the depth or spontaneity of disclosure compared with in‐person interviews. Participants' physical environments and access to private space may also have influenced the interview situation. In the small‐group interviews, interaction enabled participants to build on shared experiences, but it may also have encouraged agreement, limited the expression of divergent or sensitive accounts, or allowed some voices to become more prominent. The use of both individual and small‐group formats should therefore be considered when interpreting the character of the accounts generated.

### 6.5 Impact and Recommendations

#### 6.5.1 Problem Addressed and Knowledge Contribution

This study addresses a persistent gap in healthcare workforce research: a limited understanding of how nursing students construct perceptions of workplace attractiveness during clinical placements and how these perceptions shape future career orientations. Although existing research on organisational attractiveness in healthcare has predominantly focused on employed nurses, the present study demonstrates that key evaluations relevant to recruitment, retention, and sustainability are formed already during clinical education. By foregrounding nursing students' experiences, the study contributes novel insight into workplace attractiveness as a dynamic and relational process that unfolds prior to labour market entry.

The findings show that workplace attractiveness, as experienced and articulated by nursing students during clinical placements, emerged as something actively produced and negotiated through everyday interactions, positioning, and access to participation in practice. Clinical placements thus emerge as a critical site where students assess not only their learning environments, but also whether particular organisational contexts appear viable, meaningful, and sustainable to imagine returning to as future workplaces.

#### 6.5.2 Theoretical Impact

This study advances a relational and processual understanding of workplace attractiveness in healthcare by shifting attention from organisational features and employer‐driven narratives to how attractiveness is constructed through everyday interactions, positioning, and participation during clinical education. In line with calls to move beyond structural workforce indicators and attend to how healthcare work is organised and experienced in practice (Kuhlmann et al. 2017; World Health Organization 2020), the findings demonstrate that structural conditions alone are insufficient for explaining how workplaces become attractive to future professionals.

Theoretically, the study extends research on organisational attractiveness in healthcare (Karjalainen et al. 2025) by demonstrating that evaluative processes relevant to recruitment and retention begin prior to labour market entry. Workplace attractiveness emerged not as a stable organisational attribute, but as a situational and relational judgement formed through early socialisation processes in clinical placements. By integrating perspectives on social identity (Ashforth and Mael 1989), social belonging (Baumeister and Leary 1995; Walton and Cohen 2007), and workplace learning and participation (Billett 2016), the study conceptualises workplace attractiveness as emerging at the intersection of identity alignment, affective inclusion, and access to meaningful participation in professional practice. This integration positions clinical education as a critical theoretical site for understanding how future‐oriented assessments of professional fit, sustainability, and belonging are formed.

#### 6.5.3 Practical and Professional Impact

From a practical perspective, the findings have implications for healthcare organisations, educational institutions, and policymakers concerned with education, competence development, and long‐term workforce supply in healthcare. The study highlights clinical placements as pivotal arenas where perceptions of workplace attractiveness are formed, underscoring that everyday practices of inclusion, recognition, and guidance are not merely educational concerns but strategic mechanisms influencing future recruitment, retention, and workforce sustainability.

For clinical educators and supervisors, the findings highlight the importance of a well‐structured onboarding process that begins before placement and continues throughout the learning period. Students benefit from clear information about schedules, roles, routines, learning goals, and practical arrangements before their first day, reducing uncertainty and allowing them to focus on learning and participation. Continuity is strengthened when students are assigned a dedicated primary supervisor who provides consistent guidance, while access to additional supervisors ensures support, shared expertise, and continuity when needed. Regular opportunities for feedback, reflection, and dialogue are essential, as they help students make sense of experiences, develop role clarity, and build confidence. Active inclusion in the wider team, a welcoming learning climate where questions are encouraged, and opportunities to progressively assume responsibility at an appropriate pace support both professional development and a sense of belonging. Together, these measures operationalise the processes of becoming a professional and becoming part of a workplace community.

For nurse managers, the findings emphasise the need to create organisational conditions that enable high‐quality supervision and learning. This includes ensuring that supervisors are formally appointed, adequately prepared for their role, and given sufficient time, resources, and managerial support to provide effective supervision. Organisations need clear onboarding structures, shared responsibility across the team, accessible learning resources, and established routines for follow‐up and continuous improvement. Managers also have a key role in fostering a welcoming and inclusive workplace culture where students feel valued, supported, and able to participate in the community of practice. At the same time, educational goals should remain the primary focus during placements, ensuring that workforce recruitment considerations do not overshadow students' learning needs. These recommendations are derived from students' experiences; their direct effects on recruitment and retention were not examined in the present study.

For educational institutions, the findings highlight the importance of preparing students not only for clinical competence, but also for navigating organisational cultures and reflecting on professional sustainability. Structured reflection on clinical placement experiences may support students in articulating and critically examining emerging perceptions of workplace attractiveness, rather than leaving such evaluations implicit or individualised. Strengthening collaboration between educational institutions and healthcare organisations may further enhance the quality of clinical placements as sites for both learning and professional socialisation.

At a policy level, the study underscores the need to align workforce strategies with the relational and social conditions under which future professionals encounter healthcare work. Rather than relying solely on structural reforms or employer branding initiatives, policies aimed at improving recruitment and retention may benefit from recognising clinical education as a formative stage where perceptions of work, identity, and sustainability are actively shaped. Although situated in a Swedish context, the findings are relevant to healthcare systems internationally, as challenges related to nursing recruitment, retention, and sustainable working conditions are widely shared across settings.

## 7 Conclusion

Understanding workplace attractiveness as something that emerges in the interaction between individuals and organisations, rather than as a fixed attribute, marks an important shift in perspective. Attractiveness becomes not a unified promise or end goal, but a process that can be strengthened or diminished through everyday relational practices, opportunities for participation, and experiences of recognition during clinical placements. It is in the interplay between students' everyday experiences during clinical placements and organisational responses that workplace attractiveness takes shape.

By integrating theories of social identity, belonging, and workplace learning, this study contributes to a more holistic and practice‐near understanding of what makes healthcare work attractive from the perspective of those entering the field. Workplace attractiveness is thereby positioned as a dynamic and culturally embedded process shaped through social relations, participation, and meaning‐making in everyday clinical placements.

## Author Contributions

**Åsa Tjulin:** conceptualization, investigation, funding acquisition, writing – original draft, methodology, validation, visualization, writing – review and editing, formal analysis, project administration, resources, data curation, supervision. **Julia Cederlund:** investigation, writing – review and editing, methodology, validation, formal analysis. **Åsa Chaikiat Ståhl:** conceptualization, funding acquisition, writing – review and editing. **Emma Brulin:** conceptualization, funding acquisition, writing – review and editing, methodology, validation. **Bodil J. Landstad:** conceptualization, funding acquisition, methodology, writing – review and editing. **Maritha Jacobsson:** conceptualization, funding acquisition, writing – review and editing, methodology. **Maria Härgestam:** conceptualization, funding acquisition, writing – review and editing. **Ann Rudman:** conceptualization, investigation, funding acquisition, methodology, validation, visualization, writing – review and editing, formal analysis.

## Funding

This study was supported by AFA Försäkring, Sweden (grant no. 20240044).

## Ethics Statement

Ethical approval for this study was obtained from the Swedish Ethical Review Authority, reference number (Dnr 2024‐05983‐01). All participants received written and verbal information and provided informed consent prior to participation.

## Conflicts of Interest

The authors declare no conflicts of interest.

## Data Availability

The data that support the findings of this study are not publicly available due to Swedish ethical legislation (the Ethical Review Act, SFS 2003:460) and the conditions of the ethical approval, which prohibit sharing of identifiable research data. Data are available from the corresponding author upon reasonable request and subject to approval by the Swedish Ethical Review Authority.
